# Induction of AML Preleukemic Fusion Genes in HSPCs and DNA Damage Response in Preleukemic Fusion Gene Positive Samples

**DOI:** 10.3390/antiox10030481

**Published:** 2021-03-18

**Authors:** Pavol Kosik, Matus Durdik, Milan Skorvaga, Daniela Klimova, Dominika Kochanova, Zlatica Cerna, Miroslav Kubes, Marek Holop, Igor Belyaev

**Affiliations:** 1 Department of Radiobiology, Cancer Research Institute, Biomedical Research Center, Slovak Academy of Sciences, 845 05 Bratislava, Slovakia; matus.durdik@savba.sk (M.D.); milan.skorvaga@savba.sk (M.S.); daniela.klimova@fmed.uniba.sk (D.K.); dominika.kochanova@savba.sk (D.K.); cerna.zlatkaa@gmail.com (Z.C.); igor.beliaev@savba.sk (I.B.); 2 Institute of Medical Biology, Genetics and Clinical Genetics, Comenius University in Bratislava, 811 08 Bratislava, Slovakia; 3 Stem Cell Lab, BIOM-R, Ltd., 841 05 Bratislava, Slovakia; kubes@biom-r.com (M.K.); holop@biom-r.com (M.H.)

**Keywords:** leukemia, preleukemic fusion genes, DNA damage, ionizing radiation, hematopoietic stem progenitor cells

## Abstract

Preleukemic fusion genes (PFGs) occurring after DNA damage in hematopoietic stem progenitor cells (HSPCs) in utero often represent the initial event in the development of childhood leukemia. While the incidence of PFGs characteristic for acute lymphoblastic leukemia (ALL) was relatively well examined by several research groups and estimated to be 1–5% in umbilical cord blood (UCB) of healthy newborns, PFGs that are relevant to acute myeloid leukemia (AML) were poorly investigated. Therefore, this study is focused on the estimation of the incidence of the most frequent AML PFGs in newborns. For the first time, this study considered the inducibility of AML PFGs in different subsets of UCB HSPCs by low-dose γ-rays and also compared endogenous DNA damage, apoptosis, and reactive oxygen species (ROS) level between UCB samples containing or lacking AML PFGs. We found that: (i) the incidence of AML PFGs in UCB was 3.19% for RUNX1-RUNX1T1, 3.19% for PML-RARα, and 1.17% for KMT2A-MLLT3, (ii) 50 cGy of γ-rays did not induce RUNX1-RUNX1T1, PML-RARα, or KMT2A-MLLT3 PFGs in different subsets of sorted and expanded HSPCs, and (iii) the AML PFG^+^ samples accumulated the same level of endogenous DNA damage, as measured by the γH2AX/53BP1 focus formation, and also the same ROS level, and apoptosis as compared to PFG^−^ controls. Our study provides critical insights into the prevalence of AML PFGs in UCB of newborns, without the evidence of a specific HSPC population more susceptible for PFG formation after irradiation to low-dose γ-rays or increased amount of ROS, apoptosis and DNA damage.

## 1. Introduction

Acute leukemia is defined as a malignant clonal expansion of progenitor cells coupled with a differentiation arrest. While acute lymphoblastic leukemia (ALL) represents about 85% of childhood acute leukemia with a peak in incidence between three and five years, acute myeloid leukemia (AML) is a disease of the elderly and it currently represents about 15% of newborn acute leukemia [1]. The chromosomal translocations resulting in preleukemic fusion genes (PFGs) and occurring in hematopoietic stem progenitor cells (HSPCs) often represent the initiating event leading to leukemia [2]. The genes that are involved in the PFGs frequently encode transcription factors, cell cycle regulators, or signal transduction molecules, and they play key roles in the development and function of lymphoid and myeloid cells. The most frequent chromosomal translocations/PFGs observed in the AML patients are t(8;21) RUNX1-RUNX1T1, t(15;17) PML-RARα, and rearrangements of the KMT2A gene as t(9;11) KMT2A-MLLT3 [3]. For ALL, the most considered PFGs/chromosomal translocations are ETV6-RUNX1 t(12;21), KMT2A/AFF1 t(4;11), and BCR-ABL t(9;22) [4].

Several studies reported the presence of PFGs in healthy newborns and assumed that they originate in utero [5]. While the incidence of the most frequent ALL PFGs in newborns was generally defined by various research groups to be 1–5% [6,7,8,9,10], only few and so far not consistent data are available on the AML PFG incidence. In particular, the incidence of RUNX1-RUNX1T1 was estimated to be within a very wide range, between 0.2% and 40% [9,11,12]. Such variability was accounted for various factors, including age, ethnicity, and methodological issues [5]. The incidence of PML-RARα was analyzed in only one study, providing a very high value up to 70% [11]. To the best of our knowledge, no study was performed for the estimation of KMT2A-MLLT3. In general, the available studies reported higher incidence of AML PFGs in healthy newborns as compared to ALL PFGs. In contrast, the prevalence of ALL over AML is a well-established phenomenon, requiring further validation of AML PFG incidence or explanation of this inconsistency between the ratio of AML/ALL PFGs from one side and the ratio of AML/ALL incidence from another one.

Despite the purported difference in AML and ALL PFGs, the generally estimated incidence of both ALL and AML PFGs in newborns is much higher than the prevalence of the overt childhood leukemia [13]. In order to overcome this eventual inconsistency, it was suggested that a specific population of HSPCs with PFGs occurring within a limited time window of fetal development may escape the immune system response and by the accumulation of other mutations finally leading to leukemia [14]. While most studies were looking for PFGs in mononuclear cells (MNC) of umbilical cord blood (UCB) or peripheral blood (PB), investigation of PFGs in sorted HSPC populations, besides one study on ALL relevant BCR-ABL [15], has not been performed yet. Therefore, the current study was designed toward the estimation of specific HSPC populations, which could be an ultimate source of AML PFGs and that way prevent a relatively frequent complication occurring after UCB transplantation, the donor cell leukemia (DCL) [16]. DCL is associated with the presence of the preleukemic clone in UCB graft. Hence, one of the aims of this study was the implementation of a screening test, to ensure the safety of the UCB grafts.

The presence of PFGs in many cases of secondary AML, after chemotherapy or radiotherapy of primary cancer, suggested an efficient induction of PFGs by chemotherapeutics and ionizing radiation (IR) [17,18,19,20,21,22]. This evidence was supported by experimental data considering the exposure of cell lines to IR at high doses or chemotherapeutics [23,24]. While high doses of IR are assumed as a possible factor inducing PFGs, the influence of IR at low doses has not yet been studied. On the other hand, the human population is currently exposed to low doses of IR not only from a variety of natural but especially anthropogenic sources, which are widely used in medical diagnostics or airport controls [25]. Epidemiological studies showed that atomic bomb survivors [26] and persons often undergoing IR diagnostics, such as mammography or computer tomography (CT), had a higher risk to develop leukemia [27,28,29]. Therefore, in this study, we addressed the question of whether the most frequent AML PFGs are induced by the low dose IR.

Genetic instability, which is observed in leukemia and many other cancers, has been attributed to the accumulation of reactive oxygen species (ROS) [30]. The capability of ROS to induce DNA double-strand breaks (DSBs) stresses the potential of ROS for triggering PFGs [31]. Supplementary to the ROS mediated mutagenesis reported in several studies [31,32,33], it was shown that the PFG expression may also interfere with DNA repair [34,35,36]. Significantly increased level of constitutive DSBs as measured with γH2AX/53BP1 foci in cells from ALL patients harboring BCR-ABL or ETV6-RUNX1 in comparison to patients without PGFs was reported [37]. However, the possible connection between AML PFGs and DSBs has not been investigated so far.

In this study, for the first time, we examined the incidence of RUNX1-RUNX1T1, PML-RARα, and KMT2A-MLLT3 PFGs in UCB cells, and also in sorted and expanded UCB HSPC populations of healthy Slovak newborns. Next, we analyzed the induction of AML PFGs by 50 cGy of γ-rays in different HSPC populations. Finally, because PFGs are caused by DSBs and they may correlate with genetic instability and oxidative stress, we also compared the levels of DNA damage, apoptosis, and ROS in samples carrying or lacking AML PFGs.

## 2. Materials and Methods

### 2.1. Cells

Mononuclear cells were extracted from UCB and then frozen in liquid nitrogen, as previously described [38]. The cells were stored in nitrogen as either cell pellets or resuspended in freezing medium with DMSO. Cell pellets were mainly used for estimating the incidence of AML PFGs in healthy newborns, while the in medium frozen cells were exploited to measure DNA damage, apoptosis, ROS level, and PFG inducibility. Mesenchymal stem cells from umbilical cord were provided by Eurocord-Slovakia. E. coli DH5alpha cells were kindly provided by Dr. G.P. Margison (University of Manchester, Manchester, UK). Plasmid pUC18 was purchased from ThermoFisher Scientific (Waltham, MA, USA). Details can be found in the Supplemental Methods S1.

### 2.2. Cell Irradiation

The cells were irradiated on ice by γ-rays in the dose of 50 cGy using a THERATRON Elite 80 accelerator (MDS Nordion, Ottawa, Canada). During irradiation, the cells were in 25 mL tissue culture flasks (TPP, Trasadingen, Switzerland) in a concentration of 2 × 10^6^ cells/mL. The irradiated cells were briefly warmed to 37 °C in a water bath and then incubated at 37 °C in a CO_2_-incubator for 1 h. The sham-irradiated control cells were concurrently subjected to the same manipulations as irradiated ones.

### 2.3. Cell Sorting

For each cell sorting, we used approx. 40 million MNCs. Subsequently, populations of HSPCs were sorted using BD FACS Aria (BD biosciences) into separate tubes with 200 µL of complete RPMI media in each (Supplemental Figure S1), as we previously described [15]:

- B-lymphocytes (Po1): Lin^−^ CD45^+^ CD34^−^ CD19^+^;
- nuclear non-specify lineage negative cells (Po2): CD45^+^ Lin^−^ CD34^−^ CD19^−^;
- HSPCs (Po3): Lin^−^ CD45^+^ CD34^+^ CD19^−^;
- Pre-Pro B cells (Po4): Lin^−^ CD45^+^ CD34^+^ CD19^+^;
- progenitors (Po5): Lin^−^ CD45^+^ CD45RA^−^ CD34^+^ CD38^+^;
- hematopoietic stem cells/multipotent progenitors (HSCs/MPPs) (Po6): Lin^−^ CD45^+^ CD45RA^−^ CD34^+^ CD38^−^;
- HSCs (Po7): Lin^−^ CD45^+^ CD45RA^−^ CD34^+^ CD38^−^ CD90^+^; and,
- MPPs (Po8): Lin^−^ CD45^+^ CD45RA^−^ CD34^+^ CD38^−^ CD90^−^.

The purity of cell sorting was at least 95%. In addition, three-million of MNCs were taken for analysis before sorting. Also see the Supplemental Methods S1.

### 2.4. Cell Expansion

The cell expansion of rare HSPC populations was done, as we previously described [15]. Briefly, the sorted cell populations were seeded into 24-well plates in a volume of 1.5 mL of complete expansion media. Mesenchymal stem cells from umbilical cord in the amount of 2 × 10^4^ mL^−1^ αMEM were added as “feeder” cells into each well 48 h before setting the expansion. The optimal time for harvesting was chosen before the expanded cells reach the “plateau” phase during 10–12 days of expansion, when the multiplication is sufficient and the cells still contain the optimal amount of RNA. Details can be found in the Supplemental Methods S1.

### 2.5. RNA Isolation and cDNA Synthesis

The total RNA was isolated with RNAzol (Research Molecular Center, Cincinnati, OH, USA) or innuPREP DNA/RNA columns (Analytik Jena, Jena, Germany) using the standard protocol recommended by the manufacturer [14]. The RNA yield was measured by Nanodrop (Thermo Scientific, St. Leon-Rot, Germany). cDNA was synthesized by the reverse transcription of total RNA in a standard reaction containing 1 µg of total RNA following the manufacturer’s protocol (Thermo Scientific) and then used as template for real time quantitative PCR (RT-qPCR).

### 2.6. Real Time Quantitative PCR

The RT-qPCR was performed, as previously described [14]. We addressed both specificity and sensitivity of our RT-qPCR assay in our previous studies [6,14]. Briefly, the RT-qPCR contained 2 µL cDNA (equivalent to 150 ng RNA), 300 nM each primer (VBC-Biotech, Wien, Austria), 200 nM probe (5′-fluorophore was FAM, 3′-quencher was BHQ1; (Merck, Darmstadt, Germany)), and HOT FIREPol Probe qPCR mix (Solis BioDyne, Tartu, Estonia). The plasmid standards with individual fusion genes were synthesized by Ipsogen (Qiagen, Marseille, France). The protocol of RT-qPCR, the primers, and the probes were designed according to Gabert et al. [39] RT-qPCR was performed on a BioRad CFX96 or an Aria MX instrument. All of the samples were run in triplicate and regarded as positive if at least one of three tested tubes was positive.

### 2.7. Validation of PFG Positive RT-qPCR Product by Standard PCR and Sequencing

The RT-qPCR product was digested with Exo/Sap (Affymetrix, Santa Clara, CA, USA) to remove contaminating primers, the TaqMan probe, and free dNTPs, which may interfere with subsequent sequencing [14]. The RT-qPCR product was first re-amplified in standard PCR using primers that contained restriction sites allowing directed subcloning of the PCR product into the sequencing vector pUC18. After the subcloning step and transformation in competent DH5α cells (RbCl^+^), recombinant plasmid DNA was isolated and employed as a template in the sequencing reaction while using sequencing primers of the vector.

### 2.8. Evaluation of DNA DSBs and γH2AX Pan-Staining by Imaging Flow Cytometry

AML PFG positive and negative samples that were specified for the enumeration of DNA repair foci were processed essentially as previously described [40]. Briefly, the cells were fixed for 10 min. in 1 mL of cold 3% paraformaldehyde. The next day, cells were rehydrated, the cell pellets were resuspended in 100 µL BSA PBS with a specific combination of antibodies for 53BP1 (anti-53BP1 rabbit (Novus, Cambridge, United Kingdom) and secondary Alexa Fluor 488 anti-rabbit, in dilution 1:1000 and 1:300, respectively) and γH2AX (AF488 conjugated anti-γH2AX antibody (BD biosciences, San Jose, United States), in dillution 1:25) proteins. CD34-APC conjugate (Miltenyi Biotec, Bergish-Gladbach, Germany) in concentration 1:30 was used for staining of CD34^+^ HSPCs. From each sample, minimum 10,000 cells were captured while using the ImageStreamX-100 (Amnis, Seattle, WA, USA). The data were analyzed using Inspire software, as previously described [6,41]. Briefly, single cells were gated according to the area and circularity in the brightfield channel. Next, the sharp cell images were chosen using gradient RMS feature. White blood cells were gated from sharp cells based on DAPI fluorescence. Subsequently, DAPI-positive cells were divided into CD34^+/−^ populations. From both populations, cells with whole nucleus γH2AX staining (known as γH2AX pan-staining), which is believed to appear in the early and late stages of apoptosis [42,43,44,45], were gated after visual inspection of cells and γH2AX foci were analyzed in the rest of the cells.

### 2.9. ROS

For analysis of ROS, the samples were processed essentially, as previously described using Cell ROX Green kit (Life technologies, Grand Island, NY, USA) [46]. Briefly, AML PGF positive and negative samples were simultaneously thawed, incubated 3 h, and one million cells were taken for ROS measurement. Subsequently, Cell ROX solution and antibodies against surface markers were added, specifically CD45-V450 (BD biosciences) for lymphocyte cells, CD34-APC (Myltenyi Biotec, Bergisch Gladbach, Germany) for HSPCs, and 7-AAD (BD biosciences, San Jose, CA, USA) for dead cells. After 45 min. of incubation, the samples were analyzed by ImageStreamX-100 (Amnis). The data were analyzed via Inspire software. For positive control, cells were treated with Tert-butyl hydrogen peroxide (200 µM).

### 2.10. Quantification and Statistical Analysis

Statistical analysis was carried out by Statistica 8.0 software (Dell software, Round Rock, TX, USA). The data were analyzed by ANOVA with Fisher LSD and the results were considered to be significantly different at *p* < 0.05.

## 3. Results

### 3.1. Presence of AML PFGs in UCB of Newborns

**Figure 1:**
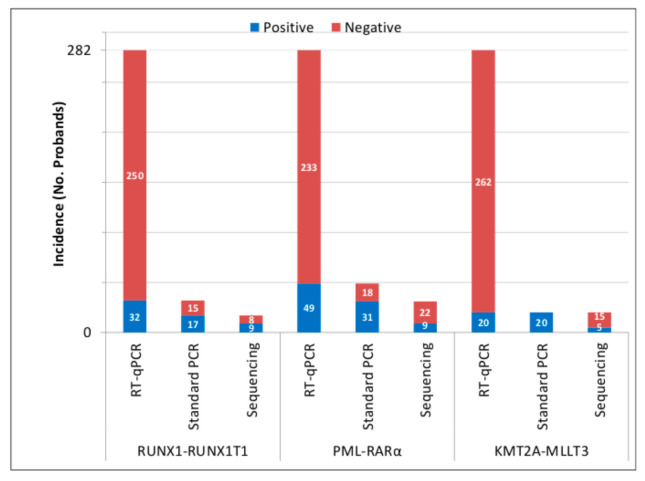
Incidence of acute myeloid leukemia (AML) preleukemic fusion genes (PFGs) in umbilical cord blood (UCB) of Slovak newborns. The figure shows the number of PFG negative and positive samples detected by real time quantitative PCR (RT-qPCR) and validated by standard PCR and sequencing.

We isolated total RNA from 282 UCB samples. The quality of RNA transcripts was checked by amplification of c-ABL gene while using RT-qPCR. The average values of c-ABL gene copies after isolation by RNAzol and RNA columns achieved 31,700 and 34,193 *per* 1× 10^5^ cells, respectively, indicating high quality of RNA. All of the samples were analyzed for RUNX1-RUNX1T1, PML-RARα, and KMT2A-MLLT3. RT-qPCR revealed the presence of RUNX1-RUNX1T1 in 32/282 (11.3%), PML-RARα in 49/282 (17.3%), and KMT2A-MLLT3 in 20/282 (7.09%) samples. After validation by standard PCR, specific bands were visualized in 17/32 (53.1%) of RUNX1-RUNX1T1, 31/49 (63%) of PML-RARα, and 20/20 (100%) of KMT2A-MLLT3 positive samples. Sequencing confirmed specific sequence in 9/17 (52.9%) of RUNX1-RUNX1T1, 9/31 (29%) of PML-RARα, and 5/20 (25%) of KMT2A-MLLT3 standard PCR positive samples (Figure 1).

In most cases, false positivity was caused by: (i) total or partial absence of one of the fusion partners, (ii) absence of the probe primer sequence used in the RT-qPCR, and (iii) in the case of PML-RARα, very long sequences of PML gene were observed (167 bp instead of 40 bp), three clones contained DDX5-RARα fusion transcript, and two clones contained the sequence of 2DHHC8 gene (supplemental Data S1). Finally, RUNX1-RUNX1T1, PML-RARα, and KMT2A-MLLT3 fusion transcripts were confirmed in 9/282 (3.19%), 9/282 (3.19%), and 5/282 (1.77%) UCB samples, respectively (Table 1, Table 2 and Table 3).

The validation rate was calculated for RUNX1-RUNX1T1, PML-RARα, and KMT2A-MLLT3 to be 28.13% (9/32), 18.3% (9/49) and 25% (5/20), respectively. The mean number of copies *per* 1 × 10^5^ cells in RUNX1-RUNX1T1, PML-RARα, and KMT2A-MLLT3 positive samples was estimated to be 2.01, 6.63, and 15.9, respectively.

### 3.2. Inducibility of AML PFGs in Different Subsets of HSPCs by 50 cGy of γ-rays

In this study, we analyzed the inducibility of the most frequent AML PFGs, namely: KMT2A-MLLT3, RUNX1-RUNX1T1, and PML-RARα in UCB HSPC populations from four newborns, whose UCB MNCs have previously been tested as PFG negative by RT-qPCR (P103, P138, P555, and P558). MNCs from IR irradiated and matched control samples of the same proband were sorted into eight different populations with the aim to establish the most susceptible population for PFG formation. Additionally, part of the MNC was taken for analysis before sorting. In some cases, we were not able to sort all eight cell populations or, even if they were sorted, we did not get enough RNA (1 µg) for reverse transcription and RT-qPCR (Supplemental Table S1). However, at least six populations were sorted from each irradiated or control MNC sample (in total 58/64 sorted populations). RT-qPCR was done on 51 of 58 sorted samples for RUNX1-RUNX1T1, on 53 of 58 for KMT2A-MLLT3, and on 49 of 58 sorted samples for PML-RARα. RUNX1-RUNX1T1, KMT2A-MLLT3, and PML-RARα transcripts were found in 11/26 (42.3%), 1/27 (3.7%), and 4/25 (16%) irradiated samples, respectively, and 10/25 (40%), 2/26 (7.7%), and 3/24 (12.5) matched control samples, respectively (Supplemental Tables S1–S4). Positive samples were further validated by standard PCR and sequencing (Supplemental Table S5). Specific bands corresponding by size to RUNX1-RUNX1T1 (∼97 bp) were visualized in 3/21 (9.4%) positive samples (2 IR exposed vs. 1 control sample). However, the sequencing data only confirmed transcripts in 2/3 standard PCR validated samples, specifically in the IR exposed MPP (P138, Po8-I, 3/3 wells, 20.1; 5.4; 11.7 copies) and control HSPC (P558, Po3-C, 1/3 wells, 1 copy) populations (Supplemental Data S2). While PML-RARα bands (∼129 bp) were visualized in 5/7 RT-qPCR products, sequencing data did not confirm the specific sequence in any of these samples (0/5). KMT2A-MLLT3 positivity tested by RT-qPCR was not confirmed by standard PCR (0/3). The validation rate for RUNX1-RUNX1T1, KMT2A-MLLT3, and PML-RARα transcripts achieved 9.5% (2/21), 0% (0/3), and 0% (0/7), respectively. To conclude, our data did not reveal cell population with a higher sensitivity to the formation of radiation-induced PFG, responsible for the genesis of AML. However, independent of irradiation, RUNX1-RUNX1T1 was only found in HSPC and MPP populations.

### 3.3. Evaluation of DNA Damage, Apoptosis and ROS in PFG Positive and Matched Negative Samples by Imaging Flow Cytometry

**Figure 2:**
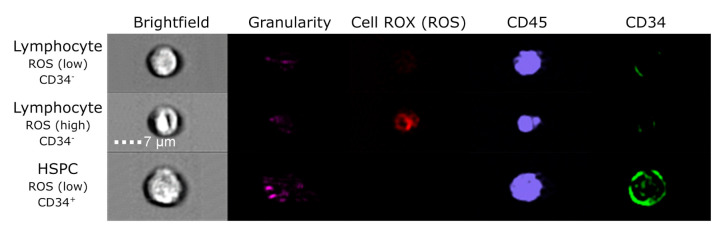
Representative images of ROS in RUNX1-RUNX1T1 PFG^+^ UCB samples analyzed by ImageStream. Brightfield as forward scatter, granularity as side scatter, Cell ROX as indicator of ROS level, CD45 antibody for lymphocytes, and CD34 antibody against hematopoietic stem progenitor cells (HSPCs) are visualized.

**Figure 3:**
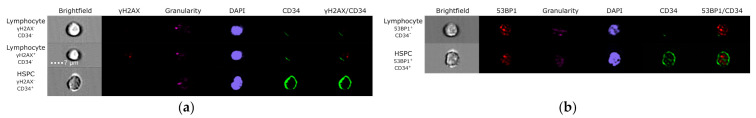
Representative images of double-strand breaks (DSBs) in RUNX1-RUNX1T1 PFG^+^ UCB samples analyzed by ImageStream. (**a**) γH2AX foci. (**b**) 53BP1 foci. Brightfield as forward scatter, granularity as side scatter, γH2AX, and 53BP1 proteins as indicators of DSBs, DAPI for DNA, and CD34 antibody against HSPCs are visualized.

We analyzed ROS (Figure 2, Supplemental Figure S2) and DSBs (Figure 3, Supplemental Figure S3) in three KMT2A-MLLT3 and three RUNX1-RUNX1T1 positive probands in order to study possible relationship between PFGs, oxidative stress and DNA damage.

**Figure 4:**
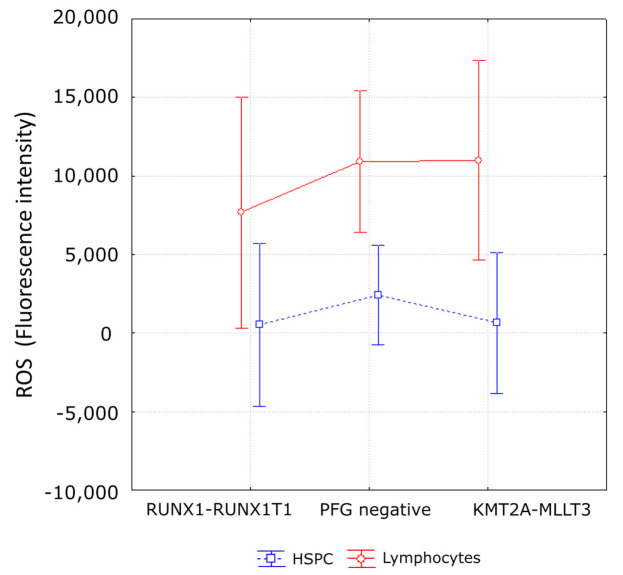
ROS levels in UCB cells of PFG^+^/PFG^−^ probands. The mean values of ROS level from 3 RUNX1-RUNX1T1 PFG^+^, 3 KMT2A-MLLT3 PFG^+^, and 3 PFG^−^ probands are shown. Lymphocytes and HSPCs are visualized separately. The error bars represent 95% confidence interval.

The matched negative control to each positive sample was chosen according to: (i) gender and (ii) time between collecting UCB and sample processing (Supplemental Table S6). Statistical analysis of all data by multifactorial ANOVA showed no effect of PFGs on ROS level (*p* = 0.57). By further analysis, we did not find a higher level of ROS in RUNX1-RUNX1T1 or KMT2A-MLLT3 positive samples when compared to control, neither in lymphocytes (Fisher LSD, *p* = 0.39, 0.91, respectively) nor in HSPCs (Fisher LSD, *p* = 0.95, 0.44, respectively). Multifactorial ANOVA also revealed the dependence of ROS production on cell type (*p* = 0.002). This dependence was caused, regardless of the presence of PFGs, by a lower ROS level in HSPCs when compared to lymphocytes (Figure 4).

**Figure 5:**
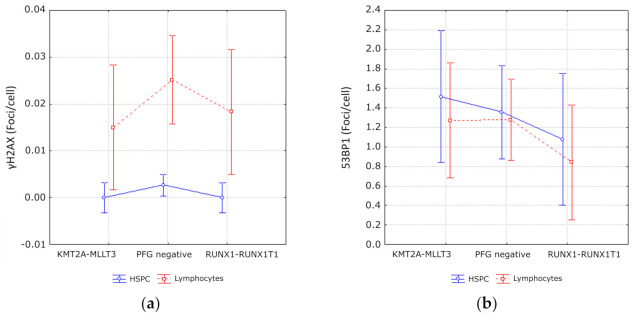
DNA repair foci in UCB cells of PFG^+^ and PFG^−^ probands. (**a**) γH2AX foci. (**b**) 53BP1 foci. Data for lymphocytes and HSPCs are presented. Mean values of foci per cell from 3 RUNX1-RUNX1T1 PFG^+^, 3 KMT2A-MLLT3 PFG^+^, and 3 PFG^−^ probands are displayed along with 95% confidence intervals.

A comparison of DNA damage based on counts of γH2AX or 53BP1 foci in RUNX1-RUNX1T1 and KMT2A-MLLT3 positive samples with matched negative controls did not show any statistically significant difference either (Fisher LSD, *p* > 0.05 for all comparisons) (Figure 5).

In line with the lower ROS level, we also observed a lower level of γH2AX foci in HSPCs as compared to lymphocytes (ANOVA, *p* = 0.000017) independent of the presence of PFGs in HSPCs. However, no statistically significant difference in 53BP1 foci was detected (ANOVA, *p* = 0.38).

**Figure 6:**
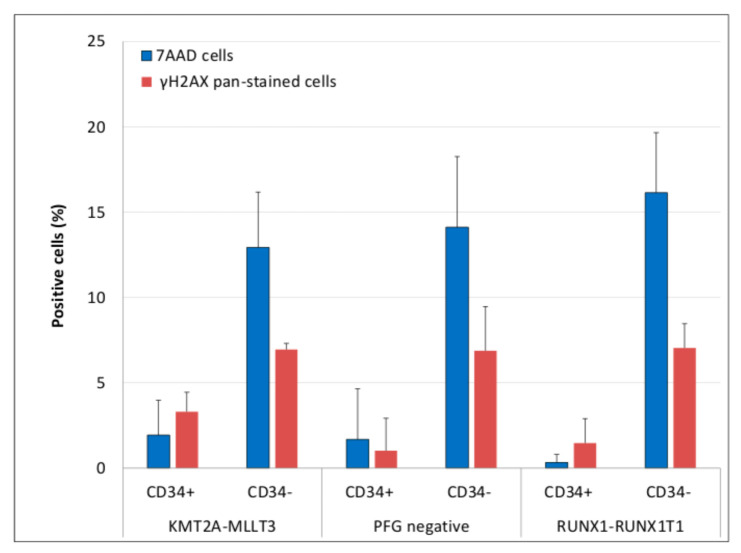
Apoptosis in CD34^+/−^ cells from PFG^+/−^ probands. The figure shows apoptosis as measured by the percentage of γH2AX pan-stained and 7-AAD^+^cells in CD34^+^HSPCs and CD34^−^ lymphocytes from PFG^+/−^ probands. Mean values from three KMT2A-MLLT3, RUNX1-RUNX1T1 and PFG negative samples are shown along with the standard deviations.

Simultaneously with analysis of ROS and DSB foci, we analyzed apoptosis while using the percentage of γH2AX pan-stained and dead 7-AAD positive cells. As in the case of ROS and DSBs, we did not observe any difference, neither in the level of γH2AX pan-stained nor 7-AAD^+^ cells between PFG^−^ and PFG^+^ samples (ANOVA, *p* = 0.46, 0.91, respectively). We also found a significantly lower level of apoptosis (γH2AX pan-staining) and cell death (7-AAD^+^ cells) in CD34^+^ HSPCs as compared with the CD34^−^ lymphocytes (ANOVA, *p* < 0.0001, 0.0001, respectively) (Figure 6).

In conclusion, our results showed no increase in the accumulation of ROS, apoptosis, or DNA damage in samples containing PFGs, although all parameters were lower in HSPCs when compared to lymphocytes.

## 4. Discussion

While the incidence and cellular origin of some ALL PFGs were examined relatively well [6,7,8,9,10,14], the most frequent AML PFGs, such as RUNX1-RUNX1T1, PML-RARα, and KMT2A-MLLT3, were only investigated sporadically, resulting in a wide range of variability, which was likely dependent on several factors including age, ethnicity and methodological issues [5]. Thus, the first aim of this study was to examine the prevalence of RUNX1-RUNX1T1, PML-RARα, and KMT2A-MLLT3 in UCB of Slovak newborns. Our data revealed the incidence of RUNX1-RUNX1T1, PML-RARα, and KMT2A-MLLT3 to be 3.19%, 3.19%, and 1.77%, respectively. The RUNX1-RUNX1T1 incidence in UCB has previously been determined as 0.2% (1/496) and 40% (63/156) by the study of Mori et al. [9] and the report of Basecke et al. [12], respectively. Interestingly, the study conducted by Mori et al. achieved 100% validation rate, when screening of all 496 samples by two methods, nested PCR and RT-qPCR, revealed the same RUNX1-RUNX1T1 positive probands. The study by Basecke et al. selected six out of 63 samples, which were tested positive by nested PCR, for validation by RT-qPCR achieving 100% validation rate, even though validation by sequencing was not performed. Leaving, without validation, the rest of 63 samples may account for large discrepancy between aforementioned studies. Our data are closer to the results of Mori et al., probably due to a larger validation in our and the Mori’s et al. study.

Song et al. [11] analyzed PML-RARα PFG in PB from healthy subjects in dependence on age. The incidence of PML-RARα in newborns, children (<25 years), and adults (>25 years) was reported to be 7/10 (70%), 10/26 (38%), and 20/38 (52%), respectively. However, the relatively small group of 73 probands was the limitation of this study. To the best of our knowledge, the induction of KMT2A-MLLT3 has not been examined so far. Thus, our current study provides a unique evidence for prenatal origin of the PML-RARα and KMT2A-MLLT3 PFGs, based on testing UCB from 282 healthy Slovak newborns. However, the incidence of PFGs that was established by us (3.19%, 3.19%, and 1.77%, respectively) highly exceeded the prevalence of overt pediatric AML. Indeed, while the incidence of pediatric AML was estimated to be about 8.1 per million person-years [47], the RUNX1-RUNX1T1, PML-RARα, and KMT2A-MLLT3 PFGs present in about 12–14%, 6–10% [3], and 6–7% [17] of total AML cases, respectively. Approximately one from ten thousand RUNX1/RUNX1T1, PML-RARα, or KMT2A-MLLT3 positive newborns will finally suffer from overt leukemia, according to these estimations. However, it might be safe to discard PFG positive UCBs from transplantation banks due to chance of abnormal proliferation or expansion of these cells upon UCB transplantation.

To the best of our knowledge, only three studies have analyzed the induction of ALL or AML PFGs by IR. In the first study, BCR-ABLs were induced in HL60 cells with X-rays at the dose of 100 Gy [24]. The second study did not show a higher level of PML-RARα after irradiation to 10 Gy of γ-rays in the lymphoid IM9 cell line than in the untreated control [48]. The third study considered three types of PFGs, namely BCR-ABL, RUNX1-RUNX1T1, and DEC-CAN [23]. In this study, HL60 and KG1 cell lines were irradiated with γ-rays at 50 and 100 Gy and then analyzed 24 or 48 h post-irradiation. While HL60 cells showed an overall poor response to IR, without a significant induction of any PFGs, IR induced RUNX1-RUNX1T1, but not BCR-ABL PFG, in the KG1 cell line. Indirect evidence of PFG induction by IR is also provided by the in vivo studies of secondary leukemia upon radiotherapy [20,21,22,49]. While aforementioned studies were performed with high doses of IR, the available epidemiological studies suggested that even low doses of IR may increase the risk of leukemia [25,26]. Primitive CD34^+^/CD38^−^ HSPCs were suggested to be leukemia-initiating cells for RUNX1-RUNX1T1, PML-RARα, and KMT2A-MLLT3 AML [50,51,52]. Moreover, the existence of specific population of HSPCs with a PFG occurring within a limited time window of fetal development was proposed to account for tremendous difference in prevalence of PFG and overt leukemia [14]. Thus, our second aim was to analyze induction of RUNX1-RUNX1T1, PML-RARα, and KMT2A-MLLT3 by the low dose of IR in various sorted and expanded HSPC populations. To the best of our knowledge, this is the first study where sorted and expanded HSPC populations were analyzed for the inducibility of PFGs by IR. Using RT-qPCR and validation by standard PCR and sequencing, we were not able to detect a higher rate of AML PFGs in samples that were irradiated with 50 cGy of γ-rays as compared to non-irradiated controls, in any of studied HSPC populations. Thus, our results indicate that other events than AML PFGs may be involved in the multistage mechanism of leukemogenesis induced by low-dose IR [25,26]. The limitation factor was the very low number of sorted HSPCs, which did not allow for including higher doses or other DSB inducing factor to the experimental design. Even though we did not observe a statistically significant increase of PFGs in irradiated samples, we detected RUNX1-RUNX1T1 only in two populations, namely MPPs and HSPCs. These data, in combination with previously published studies [23,24,48], clearly indicated that PFG formation is essentially cell type-dependent. A reason for higher susceptibility of selected populations for PFG formation could be specific mutual position of chromosomal territories and gene proximities in these cells [53]. While no data were published for AML1 and ETO proximities in MPP/HSPCs, some other genes contributing to PFGs were considered. For example, chromosomes 9 (for ABL gene) and 22 (for BCR gene) in G0 lymphocytes were located in a small, restricted volume of cell nuclei with a high probability of mutual interaction [54]. In another study, the proportion of cells, in which PML and RARA or ABL and BCR were closely associated, was higher in CD34^+^ HSPCs than in mature B-lymphoid cells [55]. Thus, we could expect that MPP or HSPC populations represent the cells where AML1 and ETO genes are at the highest proximity to form a PFG. Moreover, we also could not exclude the possibility that leukemic lesions can alter the cell of origin, so that more differentiated cells, like progenitors or MPPs, eventually show surface markers and properties of hierarchically upstream located cells [4].

The association of PFGs and leukemia with increased DNA instability through ROS up-regulation has been previously demonstrated [31,32], suggesting a common mechanism driving leukemogenesis. Therefore, our third aim was to compare DNA damage, apoptosis, and ROS accumulation between cells from AML PFG positive and negative probands. The obtained data did not confirm higher DNA damage, apoptosis, or ROS level in PFG positive samples, which supports our previously reported results with ALL PFGs [6]. There are at least three explanations for no apparent effect of PFGs on ROS, apoptosis, and DNA damage: (i) PFGs alone are not able to increase ROS, apoptosis, or DSBs in UCB cell populations; (ii) while PFGs may induce ROS, apoptosis, and DSBs, the number of PFG^+^ cells in positive probands is too low (RUNX1-RUNX1T1 (7/10^5^) and KMT2A-MLLT3 (4.4/10^5^)) for the detection of this induction; and, (iii) a secondary mutation leading to overt leukemia has to occur for genetic instability or disrupted DNA repair, which is observed in patients that suffer from leukemia with PFGs [56]. Another reason for undetected difference in ROS or DSB levels could be that none of the probands enrolled to this study have yet developed leukemia. In fact, only very small part of PFG^+^ UCB probands (0.1–1%) will get leukemia [57,58].

## 5. Conclusions

We studied incidence of the most frequent AML PFGs in UCB of Slovak newborns. RUNX1-RUNX1T1, PML-RARα, and KMT2A-MLLT3 fusion transcripts were found in 3.19%, 3.19%, and 1.77% UCB samples, respectively. We suggest discarding PFG positive UCB from transplantation banks due to a chance of abnormal proliferation or expansion of these cells upon UCB transplantation. For the first time, this study considered the inducibility of AML PFGs in sorted and expanded subsets of UCB HSPCs by low-dose γ-rays and also compared endogenous DNA damage, apoptosis, and ROS level between UCB samples from PFG^+/−^ probands. We did not reveal a cell population with a higher sensitivity to the formation of radiation-induced PFGs. However, independent of irradiation, RUNX1-RUNX1T1 was only found in HSPC and MPP populations, indicating that PFG formation is essentially cell type-dependent. Furthermore, we did not observe any difference in the amounts of ROS, DSBs, γH2AX pan-stained, and 7-AAD^+^ cells between PFG^−^ and PFG^+^ samples. One of the reasons for these results could be a low number of PFG^+^ cells in PFG positive probands.

## Figures and Tables

**Table 1:** RT-qPCR characteristics of RUNX1-RUNX1T1 positive samples validated further by standard PCR and sequencing.

No.	RUNX1-RUNX1T1 Positive Probands	No. of Positive Reactions/Triplicate	Ct (∆R)	No. of Copies/1 × 10^5^ Cells
1	P169	1/3	38.87	1
2	P174	1/3	38.29	1
3	P258	1/3	39.81	0.3
4	P267	1/3	39.64	0.3
5	P485	1/3	37.95	7.1
6	P525	1/3	40.06	1.75
7	P412	1/3	35.62	4.3
8	P278	1/3	43.3	0.4
9	P536	1/3	40	2

Abbreviations: Ct, cycle threshold.

**Table 2:** RT-qPCR characteristics of PML-RARα positive samples validated further by standard PCR and sequencing.

No.	PML-RARα Positive Probands	No. of Positive Reactions/Triplicates	Ct (∆R)	No. of Copies/1 × 10^5^ Cells
1	P417	1/3	36.11	5.7
2	P437	3/3	38.10	2.3
			37.75	2.9
			38.09	2.3
3	P461	1/3 + 2/3	37.01	4.76
			38.49	5.97
			36.57	21.4
4	P465	1/3 + 1/3	37.35	3.79
			38.14	7.5
5	P469	1/3 + 3/3	38.44	1.83
			38.96	4.3
			38.44	6.17
			37.63	10.6
6	P509	2/3	39.83	0.7
			36.99	4.8
7	P510	2/3 + 1/3	39.63	0.8
			38.12	2.6
			37.35	12.7
8	P511	2/3 + 2/3	39.27	1
			36.87	5.3
			36.91	17
			36.43	23.5
9	P525	1/3	36.25	5.2

Abbreviations are explained in Table 1.

**Table 3:** RT-qPCR characteristics of KMT2A-MLLT3 positive samples validated further by standard PCR and sequencing.

No.	KMT2A-MLLT3 Positive Probands	No. of Positive Reactions/Triplicates	Ct (∆R)	No. of Copies/1 × 10^5^ Cells
1	P293	1/3	38.59	17.5
2	P369	1/3	44.22	0.4
3	P545	1/3	39.92	3
4	P549	2/3	36.81	21
			38.05	10
5	P550	2/3	38.39	8
			35.41	52

Abbreviations are explained in Table 1.

## Data Availability

The data presented in this study are available within the article and its supplementary material. Other data that support the findings of this study are available upon request from the corresponding authors.

## Supplementary Materials

The following are available online at https://www.mdpi.com/2076-3921/10/3/481/s1, Methods S1: More detailed methods, Table S1: RUNX1-RUNX1T1, PML-RARα and KMT2A-MLLT3 PFGs in different populations of irradiated (I) and control (C) sorted HSPCs, Table S2: RT-qPCR for RUNX1-RUNX1T1 PFG, Table S3: RT-qPCR for PML-RARα PFG, Table S4: RT-qPCR for KMT2A-MLLT3 PFG, Table S5: AML PFGs revealed by standard PCR in HSPC populations sorted and expanded upon irradiation with γ-rays at 50 cGy, Table S6: Probands analyzed for ROS, apoptosis and DSBs, Data S1: Representative sequences of gene fusions found by RT-qPCR, but not confirmed by sequencing, Data S2: Representative sequences of RUNX1-RUNX1T1 fusion genes found by RT-qPCR and confirmed by sequencing, Figure S1: Gating strategy of sorting UCB cell populations, Figure S2: Positive control for analysis of ROS in KMT2A-MLLT3, RUNX1-RUNX1T1 positive and negative probands, Figure S3: Positive control for analysis of γH2AX and 53BP1 foci.
