# Supplementary material for: Induction of AML Preleukemic Fusion Genes in HSPCs and DNA Damage Response in Preleukemic Fusion Gene Positive Samples
Source: Antioxidants (Basel). 2021 Mar 18;10(3):481. doi: 10.3390/antiox10030481 (PMC8003332; doi:10.3390/antiox10030481)
Supplement: Supplementary file 1 [file antioxidants-10-00481-s001.pdf]

# Induction of AML preleukemic fusion genes in HSPCs and DNA damage response in preleukemic fusion gene positive samples

**Pavol Kosik [1],* Matus Durdik [1], Milan Skorvaga [1], Daniela Klimova [1,2], Dominika Kochanova [1], Zlatica Cerna [1], Miroslav Kubes [3], Marek Holop [3], Igor Belyaev [1]**

## Supplemental methods S1

### *Cells*

Mononuclear cells (MNCs) were extracted from UCB as previously described [1] and frozen in liquid nitrogen. The cells were stored in nitrogen either as cell pellets or resuspended in freezing medium with dimethyl sulfoxide (DMSO). Cell pellets were mainly used for estimating the incidence of AML PFGs in healthy newborns, while the in medium frozen cells were exploited to measure DNA damage, ROS level and PFG inducibility. After fast thawing of medium frozen MNCs in water bath at 37°C, the majority of adherent monocytes were removed by incubation in RPMI medium supplemented with 10% FBS, 100 IU $ml^{-1}$ penicillin, and 100 µg $ml^{-1}$ streptomycin, under standard incubation condition at 37°C in a $CO_2$-incubator.

### *Cell sorting*

Sorting of HSPCs populations was performed as we previously described [2]. Irradiated and control UCB MNC were washed in PBS (Sigma-Aldrich) and incubated 15 min in 400 µl PBS containing 1% BSA and 4 µl FC block (BD Biosciences). For compensation of fluorochrome spectral overlaps, single-color stained tubes (SCST) were prepared by adding 10 µl of cell solution to 30 µl of 1% BSA PBS with a single antibody. The rest of cell solution was used for cell sorting, where all antibodies were added. For immuno-phenotyping of different cell populations, following antibody conjugates were used: CD34-APC (Miltenyi Biotec, Bergisch-Gladbach, Germany), CD45-V450, CD38-PeCy7, CD45RA-FITC, CD90-PE, CD19-Amcyan and lineage cocktail (CD56, CD8, CD3 and CD235a-PeCy5) (all BD biosciences, San Jose, California, USA). The cells were then incubated for 60 min in the fridge at 4°C. Next, 500 µl of 1% BSA PBS was added to each tube and samples were centrifuged for 10 min at 800 rpm. Samples were resuspended in 400 µl of 1 % BSA PBS or in 200 µl for SCST. Finally, 2 µl and 1 µl of 7-AAD were added to the sample and lineage compensation tube, respectively.

Various populations of HSPCs were then sorted using BD FACS Aria (BD biosciences) into separate tubes with 200 µl of complete RPMI media (Supplemental figure S1).

## *Cell expansion*

Cell expansion of rare HSPC populations was done as we previously described [2]. The sorted cell populations were seeded on 24 well plates in a volume of 1.5 ml of complete expansion media (CEM). CEM for one well contained: (i) 1 ml of MEM alpha EAGLE including UltraGlutamine I, deoxyribonucleoside, and ribonucleosides ($\alpha$MEM), (ii) 0.5 ml IMDM including HEPES w/L-Glutamine (both LONZA) (iii) 10% of FBS (Fetal Bovine Serum, HYCLONE), (iv) 1% of ATB/ATM (Antibiotic/Antimycotic Solution, PAA) containing stem cell factor (25 ng/ml), Flt-3L (25 ng/ml), and trombopoetin (25 ng/ml) (all three R&D Systems, Minneapolis, USA). Mesenchymal stem cells (MSC) from umbilical cord in amount of $2\times10^4$/well/ml $\alpha$MEM as a "feeder" cells were added into each well 48 h before setting the expansion. Optimal starting concentration of cells for every CD34+ population was 1500 cells/well. The maximal level of expansion achieved was ~ 1,000× multiplication of starting culture. Cells were checked visually in microscope in intervals of every 2 - 4 days. On the 8th day of expansion, the cells were harvested and transferred in fresh CEM into wells with new MSC feeder. The optimal time for harvesting was chosen before the expanded cells reach the "plateau" phase during the 10 - 12 day of expansion.

**Supplemental Table S1.** RUNX1-RUNX1T1, PML-RAR$\alpha$ and KMT2A-MLLT3 PFGs in different populations of irradiated (I) and control (C) sorted HSPCs. Number of positive samples detected by RT-qPCR in triplicates is shown.

| Proband, PFG | Po1 C | Po1 I | Po2 C | Po2 I | Po3 C | Po3 I | Po5 C | Po5 I | Po6 C | Po6 I | Po7 C | Po7 I | Po8 C | Po8 I | MNC C | MNC I |
|---|---|---|---|---|---|---|---|---|---|---|---|---|---|---|---|---|
| P103, RUNX1-RUNX1T1 | ND | ND | ND | ND | 0/3 | 0/3 | 0/3 | 0/3 | 0/3 | 0/3 | 1/3 | 0/3 | 0/3 | 0/3 | ND | ND |
| P555, RUNX1-RUNX1T1 | ND | ND | 1/3 | 2/3 | 0/3 | 1/3 | 0/3 | 1/3 | 1/3 | 1/3 | 0/3 | 0/3 | 0/3 | 0/3 | 0/3 | 0/3 |
| P138, RUNX1-RUNX1T1 | 0/3 | 0/3 | 0/3 | 0/3 | ND | ND | 1/3 | 3/3 | 2/3 | 1/3 | 2/3 | 0/3 | 1/3 | 3/3[a] | 0/3 | 2/3 |
| P558, RUNX1-RUNX1T1 | 1/3 | 2/3 | 0/3 | 2/3 | 1/3[a] | 0/3 | 0/3 | 1/3 | ND | ND | 0/3 | 0/3 | ND | 0/3 | 1/3 | 0/3 |
| P103, PML-RAR$\alpha$ | ND | ND | ND | ND | 0/3 | 0/3 | 0/3 | 0/3 | 0/3 | 0/3 | 0/3 | 0/3 | 0/3 | 0/3 | ND | ND |
| P555, PML-RAR$\alpha$ | ND | ND | 1/3 | 0/3 | 0/3 | 2/3 | 1/3 | 0/3 | 0/3 | 0/3 | 0/3 | 0/3 | 0/3 | 0/3 | 0/3 | 0/3 |
| P138, PML-RAR$\alpha$ | ND | ND | ND | ND | ND | ND | 0/3 | 0/3 | 1/3 | 0/3 | 0/3 | 0/3 | 0/3 | 0/3 | 0/3 | 0/3 |
| P558, PML-RAR$\alpha$ | ND | 0/3 | 0/3 | 1/3 | 0/3 | 1/3 | 0/3 | 1/3 | 0/3 | 0/3 | 0/3 | 0/3 | 0/3 | 0/3 | 0/3 | 0/3 |
| P103, KMT2A-MLLT3 | ND | ND | ND | ND | 0/3 | 0/3 | 0/3 | 0/3 | 0/3 | 0/3 | 0/3 | 0/3 | 0/3 | 0/3 | ND | ND |
| P555, KMT2A-MLLT3 | ND | ND | 0/3 | 1/3 | 0/3 | 0/3 | 0/3 | 0/3 | 0/3 | 0/3 | 0/3 | 0/3 | 0/3 | 0/3 | 0/3 | 0/3 |
| P138, KMT2A-MLLT3 | 0/3 | 0/3 | 0/3 | 0/3 | ND | ND | 0/3 | 0/3 | 0/3 | 0/3 | 0/3 | 0/3 | 0/3 | 0/3 | 0/3 | 0/3 |
| P558, KMT2A-MLLT3 | 2/3 | 0/3 | 0/3 | 0/3 | 0/3 | 0/3 | 1/3 | 0/3 | 0/3 | 0/3 | 0/3 | 0/3 | ND | 0/3 | 0/3 | 0/3 |

Abbreviations: ND, not determined; C, control population; I, irradiated population; Po1, B-lymphocytes (Lin- CD45+ CD34- CD19+); Po2, nuclear non-specify lineage negative cells (CD45+ Lin- CD34- CD19-); Po3, HSPCs (Lin- CD45+ CD34+ CD19-); Po4, Pre-Pro B cells (Lin- CD45+ CD34+CD19+); Po5, progenitors (Lin- CD45+ CD45RA- CD34+ CD38+); Po6, HSCs/MPPs (Lin- CD45+ CD45RA- CD34+ CD38-); Po7, HSCs (Lin- CD45+ CD45RA- CD34+ CD38- CD90+); Po8, MPPs (Lin- CD45+ CD45RA- CD34+ CD38- CD90-).

[a] RT-qPCR positive samples validated by standard PCR and sequencing.

**Supplemental table S2.** RT-qPCR for RUNX1-RUNX1T1 PFG. Population, proband, number of positive reactions in triplicates/run (positivity), cycle threshold - Ct (ΔR), and number of copies / 1 × $10^5$ cells for irradiated and matched control samples are shown.

| Population | Proband, Positivity | Ct (ΔR) | Number of copies |
|---|---|---|---|
| Po1-C | P 558, 1/3 | 34.25 | 12.3 |
| Po1-I | P 558, 2/3 | 34.26; 36.11 | 12.9; 3.14 |
| Po2-C | P 555, 1/3 | 37.0 | 1.07 |
| Po2-I | P 555, 2/3 | 36.57; 34.18 | 1.36; 8.07 |
| | P 558, 2/3 | 35.9; 36.11 | 4.18; 3.6 |
| Po3-C | P 558, 1/3 | 37.84 | 0.9 |
| Po3-I | P 555, 1/3 | 37.87 | 0.51 |
| Po5-C | P 138, 1/3 | 37.91 | 1.42 |
| Po5-I | P 555, 1/3 | 36.25 | 1.73 |
| | P 138, 3/3 | 35.20; 33.28; 32.79 | 8.28; 31.1; 43 |
| | P 558, 1/3 | 36.04 | 3.6 |
| Po6-C | P 555, 1/3 | 36.35 | 1.58 |
| | P 138, 2/3 | 36.93; 36.04 | 2.8; 5.38 |
| Po6-I | P 555, 1/3 | 34.2 | 7.9 |
| | P 138, 1/3 | 37.12 | 2.45 |
| Po7-C | P 103, 1/3 | 35.26 | 3.59 |
| | P 138, 2/3 | 35.51; 32.69 | 7.2; 5 |
| Po7-I | - | - | - |
| Po8-C | P 138, 1/3 | 35.95 | 5 |
| Po8-I | P 138, 3/3 | 33.94; 35.95; 34.73 | 20.1; 5.4; 11.7 |
| MNC-C | P 558, 1/3 | 35.62 | 4.3 |
| MNC-I | P 138, 2/3 | 36.99; 36.57 | 1.69; 2.5 |

C, control population; I, irradiated population.

**Supplemental table S3.** RT-qPCR for PML-RAR$\alpha$ PFG. Population, proband, number of positive reactions in triplicates/run (positivity), cycle threshold Ct ($\Delta$R), and number of copies / 1 × $10^5$ cells for irradiated and matched control samples are displayed.

| Population | Proband, Positivity | Ct (ΔR) | Number of copies |
|---|---|---|---|
| Po2-C | P 555, 1/3 | 37.02 | 1.13 |
| Po2-I | P 558, 1/3 | 33.42 | 22.2 |
| Po3-C | - | - | - |
| Po3-I | P 555, 2/3<br>P 558, 1/3 | 37.56; 40.35<br>37.28 | 0.79; 0.14<br>2.31 |
| Po5-C | P 555, 1/3 | 37.62 | 0.7 |
| Po5-I | P 558, 1/3 | 38.27 | 1.3 |
| Po6-C | P 138, 1/3 | 38.27 | 0.5 |
| Po6-I | - | - | - |

C, control population; I, irradiated population.

**Supplemental table S4**. RT-qPCR for KMT2A-MLLT3 PFG. Population, proband, number of positive reactions in triplicates/run (positivity), cycle threshold Ct (ΔR), and number of copies / 1 × $10^5$ cells for irradiated and matched control samples are displayed.

| Population | Proband, Positivity | Ct (ΔR) | Number of copies |
|---|---|---|---|
| Po1-C | P 558, 2/3 | 37.81; 41.71 | 11.4; 1 |
| Po1-I | - | - | - |
| Po2-C | - | - | - |
| Po2-I | P 555, 1/3 | 38.24 | 3.1 |
| Po5-C | P 558, 1/3 | 39.71 | 3 |
| Po5-I | - | - | - |

C, control population; I, irradiated population.

**Supplemental table S5.** AML PFGs revealed by standard PCR in HSPC populations sorted and expanded upon irradiation with γ-rays at 50 cGy.

| Proband | Population | RUNX1-RUNX1T1 in standard PCR | PML-RARα in standard PCR | KMT2A-MLLT3 in standard PCR |
|---|---|---|---|---|
| P103 | Po7-C | Absent | ND | ND |
| P555 | Po2-C | Absent | Present[c] | ND |
| | Po2-I | Absent | ND | ND |
| | Po3-I | Absent | Present[c] | ND |
| | Po5-C | ND | Present[c] | ND |
| | Po5-I | Absent | ND | ND |
| | Po6-C | Absent | ND | ND |
| | Po6-I | Absent | ND | ND |
| P138 | Po5-C | Absent | ND | ND |
| | Po5-I | Absent | ND | ND |
| | Po6-C | Absent | Absent | ND |
| | Po6-I | Absent | ND | ND |
| | Po7-C | Absent | ND | ND |
| | Po8-C | Absent | ND | ND |
| | Po8-I | Present[b] | ND | ND |
| | MNC-C | Absent | ND | ND |
| | MNC-I | Present[c] | ND | ND |
| P558 | Po1-C | ND | ND | Absent |
| | Po1-I | Absent | ND | ND |
| | Po2-I | Absent | Absent | ND |
| | Po3-I | ND | Present[c] | ND |
| | Po3-C | Present[b] | ND | ND |
| | Po5-C | ND | ND | Absent |
| | Po5-I | Absent | Present[c] | ND |
| | MNC-C | Absent | ND | ND |
| | Total positive | 2/21 | 0/7 | 0/2 |

Abbreviations are explained in Supplemental table S1.

[b]Positive standard PCR samples confirmed by sequencing.

[c]Positive standard PCR samples not confirmed by sequencing.

**Supplemental table S6**. Probands analyzed for ROS, apoptosis and DSBs. $PFG^+$/$PFG^-$ probands, gender, and time from collecting UCB till its processing are displayed.

| **PFG positive proband** | **Matched negative control** | **Gender** | **Time till processing (positive/negative)** |
|---|---|---|---|
| P545<br>KMT2A-MLLT3 | P538 | Male | 17 h / 18 h |
| P369<br>KMT2A-MLLT3 | P374 | Female | 18 h / 19 h |
| P293<br>KMT2A-MLLT3 | P249 | Male | 20.5 h / 19.5 h |
| P412<br>RUNX1-RUNX1T1 | P407 | Male | 22 h / 20 h |
| P525<br>RUNX1-RUNX1T1 | P509 | Male | 13 h / 13 h |
| P485<br>RUNX1-RUNX1T1 | P481 | Male | 17 h / 18 h |

**Supplemental data S1.** Representative sequences of gene fusions found by RT-qPCR, but not confirmed by sequencing.

#**P296 ($PML_1$-RARA⁺),** GenBank accession number: MW557603

ccccaacagcaaccacgtggccagtCGGGTTATTCGAGTGACCGAGACCGCGGCCGGGACCGAGGCCATTG
AGACCCAGAGCAGCAGTTCTGAAGAGAT**AGTGCCCAGCCCTCCC*TCGC***cacccccctctaccccgca*

lower case regular letters - $PML_1$ forward primer sequence (complete sequence, 25/25 identity), shadow box DEAD-box helicase 5 (DDX5) gene sequence (36/36 identity) , upper case regular letters– RARA gene sequence (complete sequence, 74/74 identity), upper case **bold** letters – RARA probe sequence (complete sequence, 20/20 identity), lower case italic letters - RARA reverse primer sequence (complete sequence, 22/22 identity)

15 nucleotides of $PML_1$ gene normally present in PML1-RARA FT is substituted with 36-nt sequence from DDX5 gene

#**P354 ($PML_1$-RARA⁺),** GenBank accession number: MW557604

ccccaacagcaaccacgtggccagt**CCTATGACAGCCTGCTCAATCCTGGCTCGCCTGGTGGCCACGCCT
GCCCTGCCCACCCAGCAGTTGGCGTGGCCGGATACCACTCACCCTACCTGCATCCTGGGG
CAACGGGCGAC***tcgccaccccc-ctaccccgca*

lower case regular letters– $PML_1$ forward primer sequence (complete sequence, 25/25 identity), shadow box – zinc finger DHHC-type containing 8 (ZDHHC8) gene sequence (140/140 identity), lower case italic letters – RARA reverse primer sequence (complete sequence, 21/22)

15 nucleotides of $PML_1$ gene and 52 nucleotides of RARA gene normally present in PML1-RARA FT is substituted with 140-nt sequence from ZDHHC8 gene

**Supplemental data S2.** Representative sequences of RUNX1-RUNX1T1 fusion genes found by RT-qPCR and confirmed by sequencing.

#**P558, Po3-C (RUNX1-RUNX1T1⁺),** GenBank accession number: MW557601

**cacctaccacagagccatcaaaaTCACAGTGGATGGGCCCCGAGAACCTCGA**AATCGTACTGAGAAGCA CTCCAC*aatgccagactcacctgtggat*

**bold** letters – AML1 gene sequence (complete sequence, 52/52 identity), regular letters – ETO gene sequence (complete sequence, 45/45 identity), lower case **bold** letters – AML1 forward primer sequence, (complete sequence, 23/23 identity), lower case italic letters - ETO reverse primer sequence (complete sequence, 22/22 identity), underlined letters – AML1 probe sequence (complete sequence, 30/30 identity) #P558 3C contains wild-type RUNX1-RUNX1T1 FT sequence (100% identity)

#**P138, Po8-I (RUNX1-RUNX1T1$^{+}$),** GenBank accession number: MW557602

**cacctaccacagagccatcaaaaTCACAGTGGATGTGCCCCGAGAACCTCGA**AATCGTACTGAGAAGCA CTCCAC*aatgccagactcacctgtggat*

the sequence assignment is the same as in (3), **T** represents a single base substitution (G → T) resulting into ~ 99% identity with wild-type RUNX1-RUNX1T1 FT

**Supplemental figure S1.** Gating strategy of sorting UCB cell populations. Figure shows gating of different HSPC populations numbered from 1 to 8 (Po1-8) as described in the Materials and Methods.

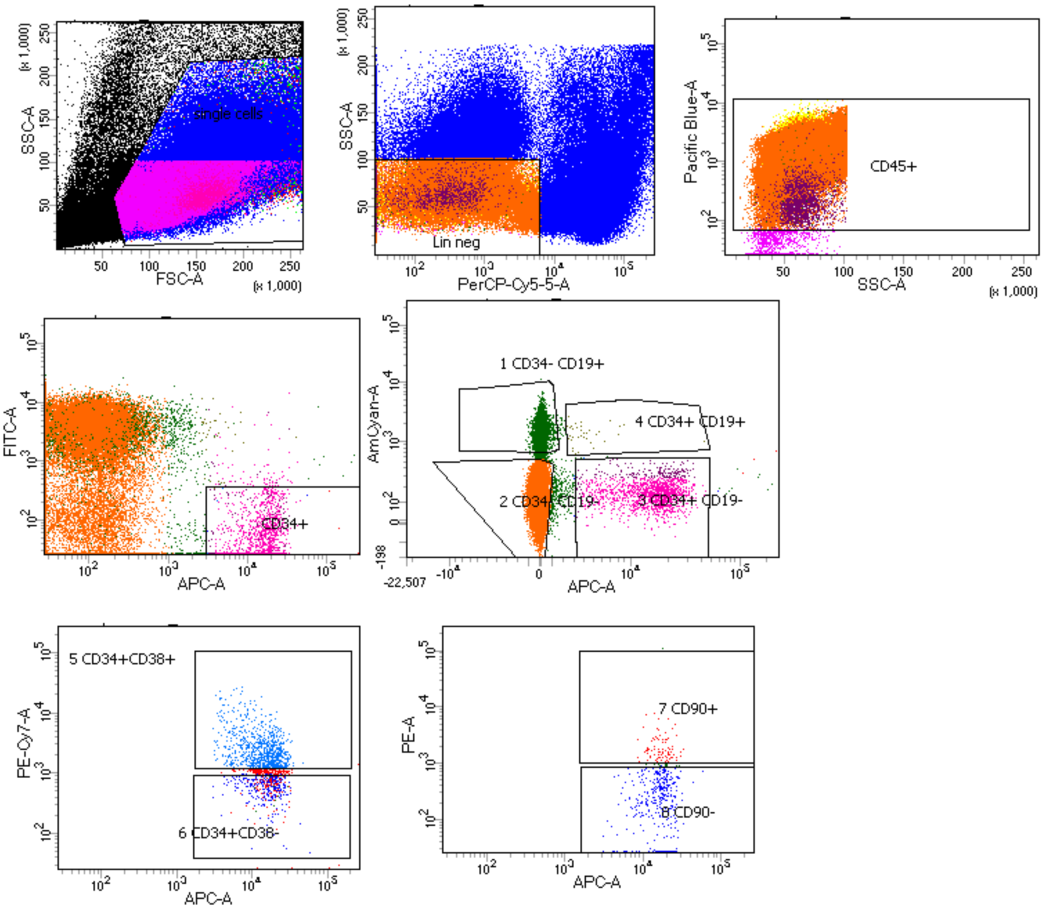

**Supplemental figure S2.** Positive control for analysis of ROS in KMT2A-MLLT3, RUNX1-RUNX1T1 positive and negative probands. 200 mM tert-butyl hydroperoxide (TBHP) was used as positive control and statistically significant increase of ROS was observed (ANOVA, $p = 0.000001$).

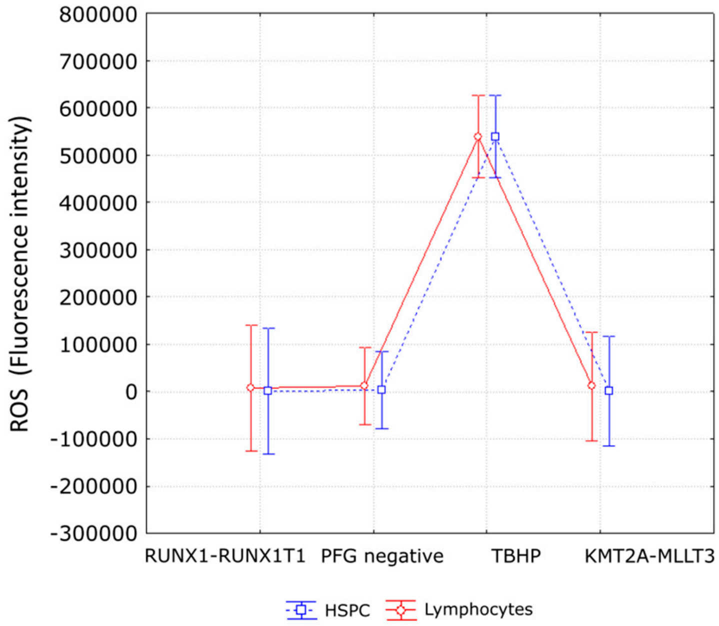


**Supplemental figure S3.** Positive control for analysis of γH2AX and 53BP1 foci. (a) γH2AX and 53BP1 foci in PFG- UCB lymphocytes 30 min after exposure to 0 and 2 Gy of γ-rays. The numbers of foci per cell are displayed. (b) Representative images of γH2AX (red) and 53BP1 (green) foci in PFG- UCB lymphocytes 30 min post-irradiation with 2 Gy of γ-rays

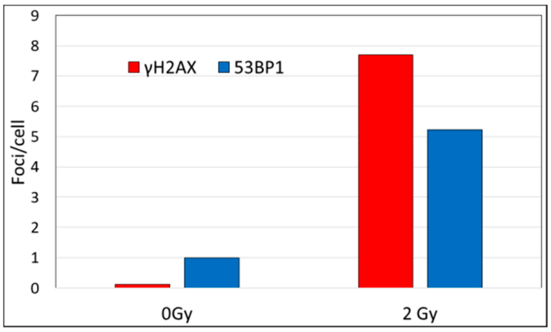


(**a**)

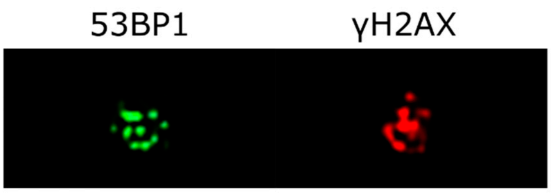


(**b**)

## REFERENCES


1. Vasilyev, S.A.; Kubes, M.; Markova, E.; Belyaev, I. DNA damage response in CD133 + stem/progenitor cells from umbilical cord blood: low level of endogenous foci and high recruitment of 53BP1. *International journal of radiation biology* **2013**, *89*, 301-309, doi:10.3109/09553002.2013.754555.
2. Jakl, L.; Skorvaga, M.; Beresova, K.; Kosik, P.; Durdik, M.; Jakubikova, J.; Holop, M.; Kubes, M.; Zastko, L.; Markova, E., et al. BCR/ABL preleukemic fusion gene in subpopulations of hematopoietic stem and progenitor cells from human UCB. *Neoplasma* **2020**, *67*, 158-163, doi:10.4149/neo_2019_190925N965.
3. Durdik, M.; Kosik, P.; Gursky, J.; Vokalova, L.; Markova, E.; Belyaev, I. Imaging Flow Cytometry as a Sensitive Tool to Detect Low-Dose-Induced DNA Damage by Analyzing 53BP1 and gamma H2AX Foci in Human Lymphocytes. *Cytom Part A* **2015**, *87A*, 1070-1078, doi:10.1002/cyto.a.22731.
